# Report of Diseases

**Published:** 1811-02

**Authors:** J. Reid

**Affiliations:** Grenville-street, Brunswick-square


### REPORT OF DISEASES,

*Under the Care of the late Senior Physician of the Finsbury Dispensary, from the 20th of December,* 1810, *to the 20th of January,* 1811.

The Reporter was a few days since consulted by letter from a remote part of the country, with regard to the expediency of a chirurgical operation in a very grievous instance of scrophulous disease. At a distance from the spot, and the case being of a nature partly surgical, it was of course only in a very qualified and conditional manner that he ventured to give his medical opinion. The circumstances and history of the complaint, however, were represented to be such, as led him to discourage the the two hasty performance of the meditated operation. Scrophula being a disease of the constitution, is seldom to be remedied by the extraction or amputation of parts. The human frame rarely indeed suffers, unless when it is induced by external violence from any morbid affection that may strictly be regarded as *local*. The appearance of it may be superficial, or confined to a particular spot, but the real root is for the most part fixed in the interior, and is secretly ramified throughout the whole substance of the frame. For want of a due regard to this circumstance, limbs may be lost without life being preserved, or health in any degree amended by the deprivation.

A case of epilepsy, that has recently fallen under the Reporter's notice, was a considerable time before anticipated, in a certain degree, by feelings which not unfrequently occur in a person who is destined, at some future period, to be the subject of this affection. Not merely an acquaintance with the actual symptoms of a disorder, bnt with the previous history also of the patient, are highly interesting and instructive: the latter knowledge is often as necessary to the prevention, as the former is to the cure, of a disease. It is of importance to know and to interpret rightly those signs which portend the approach of any formidable malady, that our fears may be aroused in time, and that we may seasonably oppose to the morbid tendency all the means of precaution and counteraction in our power. In complaints which fall under the denomination of nervous, this is more particularly incumbent. Upon minute enquiry of the patieut alluded to, it appeared that several years before the complete formation of an epileptic paroxysm, she had been liable to a sleepiness, which was not removed by actual sleep, to a frequently recurring sense of intoxication, without having taken any inebriating draught or drug, to an almost habitual unsteadiness upon the feet, and sometimes to an actual staggering. She had been also remarkable for some months before her late, which was her first attack, of this complaint, for an incessant restlessness, and propensity to locomotion, a continual disposition to change her posture or her place. This mobility extended likewise to the mind, so that a permanent direction of it to one subject was an effort beyond her power. The attention was always fluttering on the wing. Not long before her epilepsy, she mentions having frequently experienced a variety of uncomfortable feelings, such as flashes of light before her eyes, head-ach, violent rushings as it seemed of blood towards the head, dizziness, dimness and confusion of vision, and a frequent sense of faintness approaching to syncope. She also states the having been subject to transient absences of the intellectual faculty, which would seem to desert her for a few minutes, and then return in a manner that she could not account for. It is but seldom that we meet with a person whose previous life afforded so many admonitory hints of the specific danger which threatened her constitution; although perhaps it is for want of a scrutiny sufficiently strict that we do not ascertain, in every case of true epilepsy, the occurrence of most at least of these preliminray circumstances of awful presage.

J. REID.

*Grenville-street, Brunswick-square,*

